# Long-term outcome and chest pain in patients with true versus non-true bifurcation lesions treated with second-generation drug-eluting stents in the TWENTE trial

**DOI:** 10.1007/s00380-015-0786-6

**Published:** 2016-01-08

**Authors:** K. Gert van Houwelingen, Liefke C. van der Heijden, Ming Kai Lam, Marlies M. Kok, Marije M. Löwik, J. W. Louwerenburg, Gerard C. M. Linssen, Maarten J. IJzerman, Carine J. M. Doggen, Clemens von Birgelen

**Affiliations:** 1 Department of Cardiology, Thoraxcentrum Twente, Medisch Spectrum Twente, Haaksbergerstraat 55, 7513 ER Enschede, The Netherlands; 2 Department of Cardiology, Ziekenhuisgroep Twente, Almelo and Hengelo, The Netherlands; 3 Health Technology and Services Research, MIRA – Institute for Biomedical Technology and Technical Medicine, University of Twente, Enschede, The Netherlands

**Keywords:** Percutaneous coronary intervention, Resolute stent, Xience V stent, Bifurcation treatment, Newer-generation drug-eluting stents

## Abstract

The objective of this study is to assess 3-year clinical outcome of patients with *true* bifurcation lesions (TBLs) versus *non*-true bifurcation lesions (non-TBLs) following treatment with second-generation drug-eluting stents (DES). TBLs are characterized by the obstruction of both main vessel and side-branch. Limited data are available on long-term clinical outcome following TBL treatment with newer-generation DES. We performed an explorative sub-study of the randomized TWENTE trial among 287 patients who had bifurcated target lesions with side-branches ≥2.0 mm. Patients were categorized into TBL (Medina classes: 1.1.1; 1.0.1; 0.1.1) versus non-TBL to compare long-term clinical outcome. A total of 116 (40.4 %) patients had TBL, while 171 (59.6 %) had non-TBL only. Target-lesion revascularization rates were similar (3.5 vs. 3.5 %; *p* = 1.0), and definite-or-probable stent thrombosis rates were low (both <1.0 %). The target-vessel myocardial infarction (MI) rate was 11.3 versus 5.3 % (*p* = 0.06), mostly driven by (periprocedural) MI ≤48 h from PCI. All-cause mortality and cardiac death rates were 8.7 versus 3.5 % (*p* = 0.06) and 3.5 versus 1.2 % (*p* = 0.22), respectively. The 3-year major adverse cardiac event rate for patients with TBL versus non-TBL was 20.0 versus 11.7 % (*p* = 0.05). At 1-, 2-, and 3-year follow-up, 6.5, 13.0, and 11.0 % of patients reported chest pain at less than or equal moderate physical effort, respectively, without any between-group difference. Patients treated with second-generation DES for TBL had somewhat higher adverse event rates than patients with non-TBL, but dissimilarities did not reach statistical significance. Up to 3-year follow-up, the vast majority of patients of both groups remained free from chest pain.

## Introduction

True bifurcation lesions (TBLs) are characterized by an advanced atherosclerotic disease burden that obstructs at bifurcation level both the main vessel and the side-branch. Percutaneous coronary interventions (PCIs) of TBL are often technically more challenging, require more often two-stent techniques [1, 2], and have previously been associated with somewhat lower technical success rate and a higher restenosis risk [3–5]. Meanwhile, second-generation drug-eluting stents (DES) were developed, which have shown favorable outcomes in broad patient populations [6, 7]. In bifurcation lesions, the use of these contemporary DES reduced the incidence of restenosis as compared to early DES [8–11], which might partly be related to an improved side-branch access [12]. Nevertheless, the incidence of periprocedural myocardial infarction (MI) is still increased in patients with bifurcated target lesions [13–15]. This might be related to the increased procedural complexity of bifurcation stenting or the atherosclerotic disease itself, which both are generally higher in patients with TBL. Only few large randomized clinical trials have reported data on the long-term performance of second-generation DES in bifurcated target lesions [13, 14, 16]. However, these studies comprised target lesions with a variety of bifurcation types, and clinical outcome was generally reported at the group level without specifying outcome for patients with TBL versus non-TBL [13, 14]. As a consequence, long-term outcome data of patients who were treated with second-generation DES in TBL are of interest but scarce [17].

For that reason, we performed an explorative sub-study of the TWENTE trial [7, 18] in patients with bifurcated target lesions and a side-branch size of at least 2 mm, comparing the long-term clinical outcome of patients with TBL versus patients who were treated for non-TBL only. In addition, we analyzed the patient-reported chest pain to detect potential differences between patients with TBL versus non-TBL, and to assess the relation between chest pain after bifurcation stenting and hard clinical endpoints.

## Materials and methods

### Study population

The randomized TWENTE trial (ClinicalTrials.gov NCT01066650) enrolled 1391 patients between June 2008 and August 2010 without any limit for target lesion length, reference size, and number of lesions or diseased vessels to be treated. The few inclusion and exclusion criteria (no STEMI within 48 h) and details of the study have previously been reported [7, 19]. In brief, a broad and heterogeneous population of PCI patients was randomized for treatment with the zotarolimus-eluting Resolute (Medtronic Inc., Santa Rosa, CA) or everolimus-eluting Xience V stent (Abbott Vascular, Santa Clara, CA). The TWENTE trial was approved by the accredited Medical Ethics Committee Twente and complied with the Declaration of Helsinki, and study participants provided a written informed consent. The 3-year clinical outcome of the TWENTE trial population has recently been reported [18].

The present sub-study assessed patients who had bifurcated target lesions with a side-branch reference lumen diameter of ≥2.0 mm, as measured by quantitative coronary angiography (QCA). Based on the lesion classification provided by the angiographic core lab, we categorized the study population into patients with at least one TBL that involved both main vessel and side-branch (i.e., Medina classes: 1.1.1; 1.0.1; 0.1.1) versus patients with non-TBL (i.e., Medina classes: 1.1.0; 1.0.0; 0.1.0; 0.0.1) only [20].

### Coronary intervention

The interventional procedure was performed according to standard techniques, and the choice of the concomitant medication was based on routine institutional protocols and current guidelines. In bifurcated target lesions, provisional T-stenting of the side-branch was generally preferred [21]. The treatment strategy, the technique of stenting, and the decision to perform final kissing balloon inflations were left at the discretion of the operator. In general, dual anti-platelet therapy was prescribed for 1 year.

### Coronary angiographic analysis

Analysts of the angiographic core lab at Thoraxcentrum Twente, blinded to the stent type used, classified the lesion types and performed off-line quantitative coronary angiography of all cases according to current standards with the use of dedicated edge-detection software (QAngio XA version 7.1; Medis, the Netherlands) [7]. Bifurcated target lesions, according to the definition of the present sub-study, were defined as lesions at junctions of a main vessel and a side-branch that had (after administration of intracoronary nitrates and before PCI) a diameter of ≥2.0 mm by QCA.

### Assessment of clinical follow-up

The follow-up procedures of the TWENTE trial have previously been reported [7, 19]. In brief, systematic laboratory and electrocardiographic testing were performed to identify periprocedural myocardial infarction (MI). Research nurses and analysts, blinded to the treatment arm, obtained information on clinical endpoints and chest pain by the use of a medical records and a medical questionnaire or, in the absence of a response, a telephone follow-up that was based on the same questions.

Patient-reported chest pain, the principal symptom of angina pectoris and a surrogate for myocardial ischemia, was classified into scores: patients with chest pain score 0–1 were symptom free or experienced chest pain only at the very maximum level of physical exertion but were not limited in daily activities; patients with score 2 had chest pain at moderate physical effort (during moderate/normal daily activities); and patients with score 3 had chest pain at mild physical effort or even at rest [22].

### Definition of clinical endpoints

Clinical endpoints were defined according to the Academic Research Consortium (ARC) [23, 24]. *Cardiac death* was defined as any death due to proximate cardiac cause (e.g., MI, low-output failure, fatal arrhythmia). MI was defined by any creatine kinase concentration of more than double the upper limit of normal with elevated values of a confirmatory cardiac biomarker (creatine kinase MB fraction or troponin), based on the updated ARC definition of MI. Periprocedural MI (PMI) was defined as target-vessel-related MI within 48 h after PCI [23, 24]. The cardiac markers were systematically assessed with subsequent serial measurements in case of relevant elevation or complaints. *Stent thrombosis* was defined according to the ARC as definite or probable. Target-lesion failure (TLF) was defined as a composite of cardiac death, target-vessel-related MI, or clinically indicated target-lesion revascularization (TLR), and major adverse cardiac event (MACE) was defined as a composite of all-cause mortality, any MI, emergent coronary bypass surgery, or TLR [7].

Clinical event adjudication was performed by independent, external clinical event committees, organized by independent clinical research organizations (Cardialysis, Rotterdam, the Netherlands; and Diagram, Zwolle, the Netherlands). The TWENTE trial is an investigator-initiated study, supported by equal unrestricted research grants from Abbott Vascular and Medtronic. The authors are solely responsible for the study design, conducting the study, statistical analysis, and reporting of outcomes.

### Statistical analysis

Continuous variables were expressed as mean ± standard deviation (SD), and categorical data were presented as numbers and percentages. Baseline characteristics were compared using Chi-square test or Fisher’s exact test for categorical variables and Student’s *t* test for continuous variables. The time to clinical endpoint was assessed according to the Kaplan–Meier method, and the log-rank test was applied to compare the incidence of MACE in patients with TBL versus non-TBL. Confidence intervals and *p* values were two sided. Analyses were performed using SPSS 15.0 (SPSS Inc., Chicago, IL, USA). *p* values <0.05 were considered significant.

## Results

### Demographics and cardiovascular risk profile of patients

A total of 287 (20.6 %) patients of all 1391 TWENTE trial participants had bifurcated target lesions with side-branches ≥2.0 mm. Based on the Medina classification of the bifurcation lesion, patients were categorized into the TBL (*n* = 116, 40.4 %) versus the non-TBL groups (*n* = 171, 59.6 %). Patients of the two groups did not differ in demographics and cardiovascular risk profile (Table 1).

**Table 1:** Characteristics of patients with true versus non-true bifurcation lesions with side-branches ≥2 mm

	True bifurcation lesion (TBL) group (*n* = 116)	Non-true bifurcation lesion (non-TBL) group (*n* = 171)	*p*
Age (years)	65.4 ± 10.6	64.0 ± 10.4	0.26
Female gender	30 (25.9)	44 (25.7)	0.98
Diabetes mellitus	24 (20.7)	32 (18.7)	0.68
Arterial hypertension	64 (55.2)	90 (52.6)	0.67
Hypercholesterolemia	60 (54.1)	87 (51.5)	0.67
Current smoker	33 (28.4)	42 (24.6)	0.46
Family history of CAD	53 (45.7)	90 (52.6)	0.25
Previous MI	41 (35.3)	48 (28.1)	0.19
Previous PCI	22 (19.0)	29 (17.0)	0.66
Previous CABG	12 (10.3)	11 (6.4)	0.23
Clinical syndrome			0.97
Stable angina pectoris	55 (47.4)	81 (47.4)	
Unstable angina	31 (26.7)	44 (25.7)	
Non-ST elevation MI	30 (25.9)	46 (27.9)	

Values are *n* (%) or mean (±SD). Patients of the TBL group were treated for at least one TBL

*CABG* coronary artery bypass grafting, *CAD* coronary artery disease, *MI* myocardial infarction, *PCI* percutaneous coronary intervention, *TBL* true bifurcation lesion

### Lesion characteristics and interventional procedure

The lesion characteristics (other than the Medina class) were similar for both groups, with the only exception being a slightly smaller side-branch lumen diameter in the TBL group (2.3 ± 0.3 vs. 2.4 ± 0.4 mm; *p* = 0.01) (Table 2). The rate of stent postdilatation was high and similar in both groups (95.2 vs. 94.2 %; *p* = 0.57). However, as may be expected, in patients with TBL a two-stent bifurcation approach was more often performed (41.4 vs. 11.1 %; *p* < 0.01), and the total number of stents implanted and the rate of final kissing balloon inflation were higher in this group (Table 2). If two-stent technique was applied, T-stenting (61.2 %) was generally preferred above (mini-)crush (20.9 %), culotte (10.3 %), and other two-stent approaches (7.5 %). Final kissing balloon inflation was performed in 36.4 % of patients treated with the single-stent approach and in 77.6 % of patients treated with two-stent techniques.

**Table 2:** Lesion and procedural characteristics of patients with true versus non-true bifurcation lesions

	True bifurcation lesion (TBL) group (*n* = 116)	Non-true bifurcation lesion (non-TBL) group (*n* = 171)	*p*
*Lesion characteristics*			
De novo lesions	99 (85.3)	154 (90.1)	0.23
Severe calcification	21 (18.1)	35 (20.5)	0.62
At least one aorto-ostial lesion	8 (6.9)	15 (8.8)	0.57
Treated coronary vessels			
Left main	11 (9.5)	19 (11.1)	0.66
Right coronary artery	17 (14.7)	36 (21.1)	0.17
Left anterior descending artery	91 (78.4)	124 (72.5)	0.26
Circumflex artery	41 (35.3)	62 (36.3)	0.87
Medina classification			<0.01
0.1.1	39 (33.6)	0	
1.0.1	18 (15.5)	0	
1.1.1	59 (50.9)	0	
0.0.1	0	25 (14.6)	
0.1.0	0	51 (29.8)	
1.0.0	0	44 (25.7)	
1.1.0	0	51 (29.8)	
Bifurcation angle (°)	55.7 ± 22.1	62.1 ± 41.0	0.14
Longest lesion length (mm)	20.1 ± 11.1	20.1 ± 12.3	0.78
Degree of stenosis before PCI (%)	67.5 ± 13.3	67.1 ± 13.3	0.76
Residual in-stent stenosis post PCI (%)	15.2 ± 6.2	14.3 ± 6.2	0.25
Side-branch characteristics			
Lumen diameter SB before PCI (mm)	2.3 ± 0.3	2.4 ± 0.4	0.01
Degree of SB stenosis before PCI (%)	62.8 ± 13.0	65.4 ± 18.8	0.40
Longest SB lesion length (mm)	10.0 ± 6.3	10.8 ± 8.1	0.58
*Procedural characteristics*			
Number of stents per patient	2.6 ± 1.4	2.2 ± 1.2	0.01
Total stent length per patient (mm)	50.0 ± 29.8	43.4 ± 27.4	0.06
Predilatation	90 (77.6)	115 (67.3)	0.06
Stent postdilatation	111 (95.2)	161 (94.2)	0.57
Final kissing balloon inflation	65 (56.0)	67 (39.2)	<0.01
Single- versus two-stent approach			<0.01
Single-stent approach	68 (58.6)	152 (88.9)	
Two-stent approach	48 (41.4)	19 (11.1)	

Values are *n* (%) or mean (±SD) unless otherwise stated. In case of multiple target lesions with side-branches ≥2 mm, quantitative coronary angiographic data of the lesion with the most severe lumen diameter obstruction are presented. In case of multiple bifurcated target lesions, a two-stent approach was scored if applied in at least one target lesion

*PCI* percutaneous coronary intervention, *SB* side-branch, *TBL* true bifurcation lesion

### Long-term clinical outcome

Three-year follow-up was available in 286 (99.7 %) patients; 1 patient withdrew consent during follow-up. The TLR rate was low in both groups (3.5 vs. 3.5 %; *p* = 1.0) (Table 3). The rates of definite-or-probable stent thrombosis were very low; a single definite stent thrombosis occurred after 17 months in a patient with TBL. The target-vessel MI rate was 11.3 versus 5.3 % (*p* = 0.06), mostly driven by (periprocedural) MI ≤48 h from PCI (9.6 vs. 4.7 %; *p* = 0.10). All-cause mortality and cardiac death rates were 8.7 vs. 3.5 % (*p* = 0.06) and 3.5 vs. 1.2 % (*p* = 0.22), respectively.

**Table 3:** Three-year clinical outcome of patients with true versus non-true bifurcation lesions

	True bifurcation lesion (TBL) group (*n* = 115)^a^	Non-true bifurcation lesion (non-TBL) group (*n* = 171)	*p*
*Adverse clinical events*			
All-cause mortality	10 (8.7)	6 (3.5)	0.06
*Cardiac* death	4 (3.5)	2 (1.2)	0.22
Any myocardial infarction	13 (11.3)	9 (5.3)	0.06
*Target*-*vessel-related* myocardial infarction	13 (11.3)	9 (5.3)	0.06
*Periprocedural* myocardial infarction	11 (9.6)	8 (4.7)	0.10
Myocardial infarction >48 h post PCI	2 (1.7)	1 (0.6)	0.57
Target-lesion revascularization (TLR)	4 (3.5)	6 (3.5)	1.00
Emergent coronary bypass surgery	0	0	
Definite-or-probable stent thrombosis	1 (0.9)	0	0.40
*Composite clinical endpoints*			
Target-lesion failure (TLF)	19 (16.5)	16 (9.4)	0.07
Major adverse cardiac events (MACE)	23 (20.0)	20 (11.7)	0.05

Values are *n* (%)

*PCI* percutaneous coronary intervention

^a^Due to one withdrawal of consent in the true bifurcation lesion (TBL) group, the number of patients with 3-year follow-up is one lower as compared to baseline. Target-lesion failure (TLF) is a composite of cardiac death, target-vessel-related myocardial infarction, or clinically indicated target-lesion revascularization (TLR); major adverse cardiac event (MACE) is a composite endpoint of all-cause mortality, any myocardial infarction, emergent coronary bypass surgery, or TLR; periprocedural myocardial infarctions occurred during the first 48 h after an index procedure

**Fig. 1:**
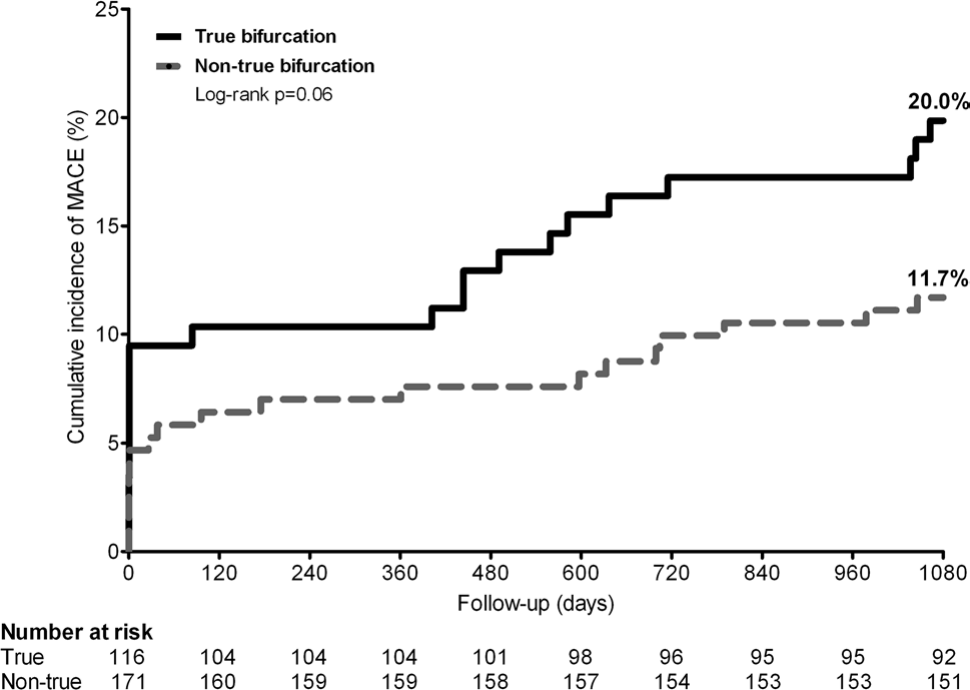
Cumulative incidence of MACE following PCI with second-generation DES in patients of the true versus non-true bifurcation lesion groups. All patients had been treated for at least one bifurcated target lesion with a side-branch ≥2 mm. *DES* drug-eluting stents, *MACE* major adverse cardiac event, a composite endpoint of all-cause mortality, any myocardial infarction, emergent coronary bypass surgery, and target-lesion revascularization, *PCI* percutaneous coronary intervention

**Fig. 2:**
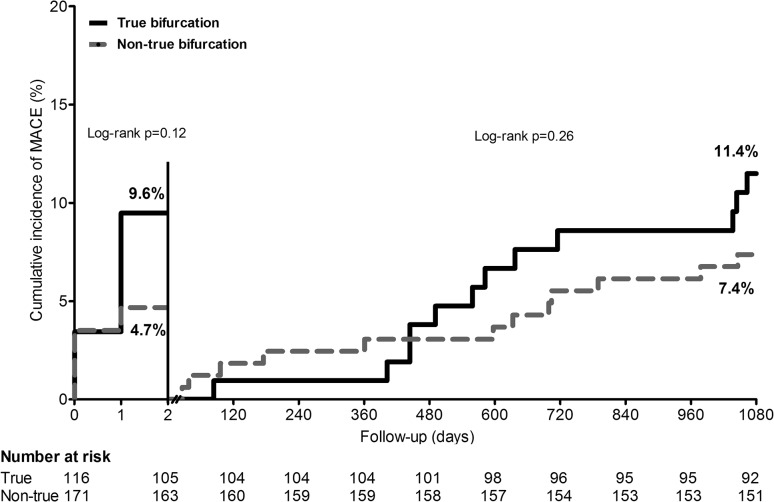
Landmark analysis of MACE at 2 days. *MACE* major adverse cardiac event, a composite endpoint of all-cause mortality, any myocardial infarction, emergent coronary artery bypass surgery, and target-lesion revascularization

The 3-year MACE rate for patients with TBL versus non-TBL was 20.0 vs. 11.7 % (*p* = 0.05), respectively. A Kaplan–Meier analysis of MACE in Fig. 1 shows the time-to-event curves, which reflect a numerically dissimilar incidence of periprocedural events and, during the second year of follow-up, a somewhat further diverging course. Landmark analysis revealed that during the first 48 h from stenting and from 48 h until 3-year follow-up, MACE was not significantly different between patients treated for TBL versus non-TBL (9.6 vs. 4.7 %, pLogrank = 0.12, and 11.4 vs. 7.4 %, pLogrank = 0.26, respectively) (Fig. 2). All but 2 MACE (related to additional non-bifurcated target lesions in patients with multivessel treatment) were related to the bifurcated target lesions.

### Chest pain at follow-up, and adverse events during consecutive time intervals

**Fig. 3:**
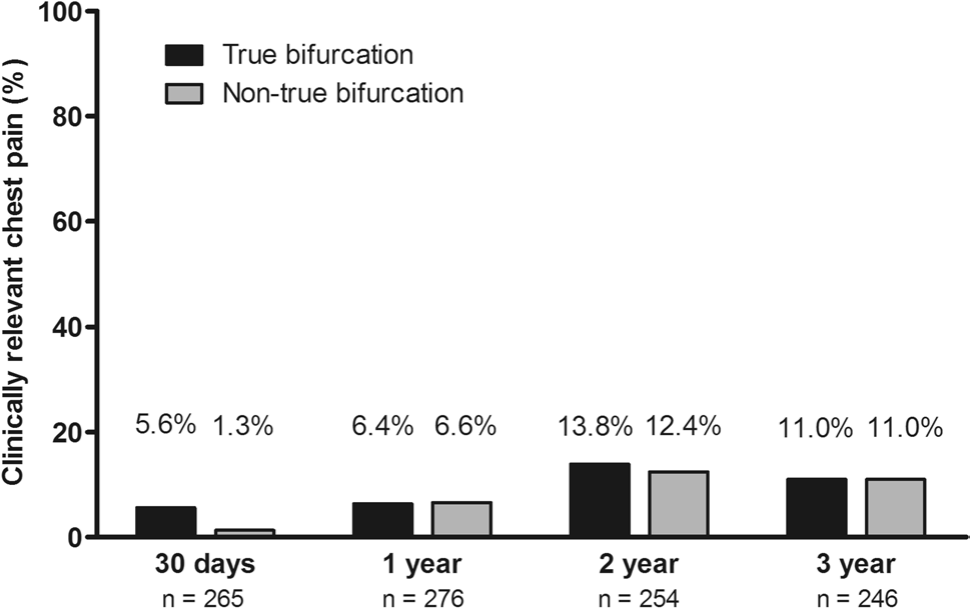
Clinically relevant chest pain at four time points of follow-up. Clinically relevant chest pain was defined as chest pain at moderate physical effort (during moderate/normal daily activities), at mild physical effort, or even at rest

At 30-day, 1-, 2-, and 3-year follow-up, overall 3.0, 6.5, 13.0, and 11.0 % of patients reported chest pain at ≤ moderate physical effort. The percentages of patients with clinically relevant chest pain at 30-day, 1-, 2-, and 3-year follow-up are presented in Fig. 3. There was no significant between-group difference in chest pain, and the vast majority of these patients were free from chest pain or had pain only occasionally at the very maximum level of physical exertion. MACE and coronary revascularization during subsequent time intervals were rare in both, patients with and without chest pain.

## Discussion

In the present study, we assessed the long-term outcome of 287 patients with bifurcated target lesions and side-branches ≥2 mm from the TWENTE trial, who were treated with second-generation DES. The 3-year TLR rate did not differ between 115 patients with TBL versus 171 patients with *non*-TBL only (3.5 % both); and in both patient groups the risk of definite-or-probable stent thrombosis was very low (both <1.0 %). During the 3-year follow-up of the present study, similar in both groups, the vast majority of patients were free from chest pain. The MACE rates of patients with TBL and non-TBL were 20 and 11.7 %, respectively. This numerical but non-significant dissimilarity in MACE (*p* = 0.05) was related to the incidence of all-cause mortality as well as MI; the latter occurred mostly within 48 h from stenting (i.e., periprocedural MI).

### Bifurcation treatment with second-generation DES

Second-generation DES have only been used in a few prospective studies that investigated clinical outcome following PCI for bifurcation lesions [9, 25–28]. In the Z-SEAside study, patients with bifurcation lesions who were treated with the Resolute stent showed a lower rate of a procedure-related composite endpoint than patients who were treated with the first-generation sirolimus-eluting stent (*n* = 75, each) [25]; and a multicenter registry of 180 patients who were treated with Resolute in bifurcated lesions also showed a low 9-month MACE rate [28]. Long-term outcome data from dedicated bifurcation studies with second-generation DES are scarce [17]. A recent pooled analysis of the 3-year clinical outcome of the randomized SEAside and CORpal studies [9, 10] showed a favorable MACE rate beyond 1 year in patients treated with Xience V stent as compared to the first-generation sirolimus-eluting stent [16]. Moreover, a retrospective study in 237 patients found acceptable clinical outcomes up to 2 years after the implantation of Xience V and Resolute in bifurcation lesions [29]. Recent sub-studies of the RESOLUTE All Comers, TWENTE, and DUTCH PEERS trials revealed similar and favorable 2- and 3-year clinical outcomes for patients who were treated with newer-generation DES for bifurcated versus non-bifurcated target lesions [13–15].

### True bifurcation stenting

Percutaneous interventions of TBL, which are characterized by an advanced atherosclerotic disease burden that obstructs the main vessel and the side-branch, are often technically more demanding, require more frequently complex techniques that involve the implantation of 2 stents, and were previously associated with a higher restenosis risk [1, 3–5]. Besides the increased procedural complexity of stenting, a more diffuse distribution of atherosclerotic plaque and a more advanced disease stage may account for an increased risk of adverse events in patients treated for TBL.

So far, only few studies address TBL treatment with second-generation DES. A small randomized study in 69 patients revealed similar angiographic and clinical 9-month results after treatment of (predominantly true) bifurcation lesions with Xience V stents, using a simple versus a complex strategy [26]. These findings were confirmed by a retrospective study in 319 patients, who were treated with Xience for TBL, showing favorable angiographic and 1-year clinical outcomes in patients treated with a 2-stent technique [30]. Despite the overall favorable outcome of bifurcation treatment with second-generation DES, there has been a higher incidence of periprocedural MI [13, 14, 27].

Our present study suggests that the risk of periprocedural MI might be higher in TBL. This could be related to the often-greater atherosclerotic burden in TBL, which may lead to more plaque displacement (with an occlusion of small side-branches) and/or distal embolization of atherothrombotic material during stenting [31]. While there is still an ongoing debate on the clinical impact of periprocedural MI [32, 33], it has recently been shown that periprocedural MI after treatment of TBL with a wide variety of DES types was associated with a significant increase in 1-year mortality [34].

In the present study, both 3-year mortality (8.7 vs. 3.5 %, *p* = 0.06) and MACE rate (20.0 vs. 11.7 %, *p* = 0.05) were, albeit statistically non-significant, numerically higher in patients with TBL versus non-TBL. While a play of chance cannot be excluded, this numeric difference might also be related to a more diffuse distribution of atherosclerosis that cannot be explained from patient demographics and cardiovascular risk factors, which did not differ between both, patients with TBL and non-TBL in the present study.

### Chest pain during follow-up of patients with bifurcation stenting

Chest pain following successful PCI with DES is clinically and economically relevant, as it often initiates the consultation of a general practitioner or cardiologist with further cardiac assessment [35]. While there is growing interest in this issue [36], most recent studies on bifurcation treatment with DES have focused on device-oriented clinical endpoints [5]. Overall, data on chest pain in patients treated with second-generation DES are scarce [5, 36]. The randomized DUTCH PEERS trial found no difference in chest pain between two DES at 1- and 2-year follow-up [22].

In the present study, we documented, similar to the DUTCH PEERS trial, patient-reported chest pain in relation to the patient’s individual range of physical activities. The majority of patients treated for TBL or non-TBL were free from clinically relevant chest pain. This symptom will generally determine whether a patient seeks further medical assessment. The absence of chest pain in the majority of our patients is supported by previous studies in bifurcation lesions, which showed that provisional stenting of the main branch generally does not result in a significant reduction in fractional flow reserve of the jailed side-branch, which means that there is usually no ischemia in the myocardium subtended by the jailed branch [37–39].

### Study limitations

Due to the explorative nature and the sample size of the present study, findings should be considered hypothesis generating. The comparison of findings from different bifurcation stent studies is hampered by the fact that there is no uniformity in the minimum size of (relevant) side-branches and no general consent on whether to determine side-branch size visually or per QCA [4, 10, 13, 14, 25, 40]. Bifurcation lesions with side-branches ≥2 mm, as addressed in our present study, were also examined in the Z-SEAside and the SEAside studies [9, 25]. Recently, advanced, user-friendly three-dimensional reconstruction and analysis software for bifurcation lesions has become available [41], but such software was not available at the time of angiographic analysis of the TWENTE trial.

## Conclusions

Patients treated with second-generation DES for TBL had somewhat higher adverse event rates than patients with non-TBL, but dissimilarities did not reach statistical significance. Up to 3-year follow-up, the vast majority of patients of both groups remained free from chest pain.
